# Longitudinal Assessment of Physical Activity and Cognitive Outcomes Among Women at Midlife

**DOI:** 10.1001/jamanetworkopen.2021.3227

**Published:** 2021-03-31

**Authors:** Gail A. Greendale, Weijuan Han, MeiHua Huang, Dawn M. Upchurch, Carrie Karvonen-Gutierrez, Nancy E. Avis, Arun S. Karlamangla

**Affiliations:** 1 Division of Geriatrics, Department of Medicine, University of California, Los Angeles; 2 Department of Community Health Sciences, University of California, Los Angeles Fielding School of Public Health; 3 Department of Epidemiology, University of Michigan School of Public Health, Ann Arbor; 4 Department of Social Sciences and Health Policy, Wake Forest School of Medicine, Winston-Salem, North Carolina

## Abstract

**Importance:**

The increasing prevalence of cognitive decline, impairment, and dementia spurs intense interest in cognitive preservation strategies.

**Objective:**

To explore the longitudinal association between physical activity (PA) and cognitive performance among women at midlife.

**Design, Setting, and Participants:**

This cohort study is an analysis from the Study of Women’s Health Across the Nation. Enrollment occurred from 1996 through 1997, and follow-up extended into 2017. Included individuals were those who had undergone cognitive measures during the first 3 cognitive test visits and had at least 1 additional cognitive measurement. Stroke prior to baseline was an exclusion, and observations were censored for subsequent stroke. Data were analyzed from June 2018 through August 2019.

**Exposures:**

Engaging in sport or exercise PA (self-reported).

**Main Outcomes and Measures:**

The Symbol Digit Modalities Test (SDMT) was used to assess cognitive processing speed. The East Boston Memory Test-Delayed (EBMT-D) was used to measure verbal episodic memory. The digit span backwards (DSB) test was used to evaluate working memory.

**Results:**

Among 1718 women with a median (range) observation time of 11.9 (0.60-13.5) years, the mean (SD) baseline age was 45.7 (2.5) years. From baseline through age 61 years, mean change in SDMT score was −0.20 annually (95% CI, −0.26 to −0.15; *P* < .001). After age 61 years, the mean change in SDMT was −0.51 yearly (95% CI, −0.54 to −0.41; *P* < .001). Beginning at age 58 years of the mean change in EBMT was −0.03 yearly (95% CI, −0.04 to −0.02; *P* < .001). Starting at age 61 years, mean (SD) change in DSB was −0.03 annually (95% CI, −0.04 to −0.01; *P* = .001). When adjusted for attrition and practice effect, PA was associated with higher concurrent SDMT and EBMT scores and a smaller decrease in SDMT score. For each unit increment in PA, there was a 0.36 increment in concurrent SDMT score (95% CI, 0.14 to 0.59; *P* = .002) and a 0.10 increment in concurrent EBMT score (95% CI, 0.05 to 0.15; *P* < .001). Greater PA was associated with a smaller annual mean decrease in SDMT score (0.06 yearly; 95% CI, 0.02 to 0.09; *P* = .001). After additional adjustment for demographic characteristics, menopause symptoms, hormone therapy use, and the presence of diabetes and hypertension, PA was not associated with trajectories (ie, levels or slopes) of any cognitive outcome.

**Conclusions and Relevance:**

This cohort study found no association between greater PA levels and cognitive outcomes among women in midlife, unlike cohort studies that begin observations at later ages, which may be associated with confounding by reverse causation (ie, cognitive decline associated with an outcome of lower PA levels).

## Key Points

**Question:**

Is physical activity during midlife associated with better performance in cognitive measures over time?

**Findings:**

This cohort study of 1718 women at midlife found that, with adjustment for socioeconomic characteristics, menopause symptoms, hormone therapy use, and presence of diabetes and hypertension, self-reported physical activity was not associated with measured cognitive performance in the domains of processing speed, verbal memory, or working memory.

**Meaning:**

These findings suggest that the cognitive protection effect of physical activity observed in later life may be an artifact of reverse causation.

## Teaser

This cohort study examines the association over time of physical activity levels and cognitive test results among women at midlife.

## Introduction

The aging of societies and increasing prevalence of cognitive decline, impairment, and dementia among older populations spur intense interest in delaying or preventing these age-associated conditions.^1,2,3^ The 2 most promising candidate cognitive preservation strategies are physical activity (PA) and hypertension control. Preventing and treating depression and diabetes may also lead to better cognitive outcomes in older age.^4,5,6,7^ However, evidence for each of these remains inconclusive.^4^ Studying the associations between cognitive decline and PA, hypertension, depression, and diabetes is made complex by the associations among those diseases themselves. This longitudinal study from the Study of Women’s Health Across the Nation (SWAN)^8^ examined the hypothesis that midlife PA may be associated with slowing of age-associated cognitive decline in the context of these chronic diseases, which are associated with PA and cognitive function.

Although there have been over 2 dozen randomized clinical trials (RCTs) of PA aimed at maintaining or improving cognitive performance in older persons (mean age of approximately 70 years across all trials), this hypothesis remains unproven. Short trial durations, variable PA interventions, and mixed assays of cognition contribute to the uncertainty.^4,9,10,11,12^ Meta-analyses, in which studies that are 26 weeks or longer predominate, have found moderate quality evidence for a beneficial effect of exercise on cognitive performance.^13,14^ These trials support that shorter-term cognitive benefits result from greater PA among older adults. However, to our knowledge, the hypothesized association between exercise and cognitive performance in middle age has been less studied. The results from 2 meta-analyses^15,16^ restricted to longer RCTs (ie, 6 months to 1 year long) did not support the postulate that PA prevents cognitive decline.

Given remaining questions about PA’s association with a cognitive benefit during midlife and over the long-term, longitudinal cohort studies may be able to play a fundamental investigative role. For example, these studies can examine exposures, such as engagement in PA over several years, that are impractical to test in an RCT. Additionally, compared with short-term PA, long-term PA may be associated with a greater improvement in cognitive performance.^4^ A meta-analysis^17^ of 21 prospective cohorts found that higher levels of PA were associated with better cognitive performance; however, the limitations of these studies must be acknowledged. Most of these studies did not employ longitudinal analyses. Moreover, most cohorts began when participants were aged 65 years or older, risking reverse causation; that is, was the decrease in PA levels an outcome associated with preclinical dementia? Assessing the association between PA and cognition earlier in the life course may avoid this pitfall.^18^

The aim of this longitudinal analysis is to explore the association between physical activity and cognitive performance in midlife among women. We hypothesized that greater levels of self-reported PA during midlife would be associated with better cognitive performance, defined as higher scores or lesser degrees of decline, with adjustments for socioeconomic factors, menopause-associated factors, and cardiometabolic comorbidity.

## Methods

Sites in SWAN obtained institutional review board approval, and participants gave written informed consent. This report follows the Strengthening the Reporting of Observational Studies in Epidemiology (STROBE) reporting guideline.

### Sample

The SWAN sample is a multisite, community-based, longitudinal, and US based cohort. Initial visits occurred in 1996 through 1997, enrolling 3302 participants.^8^ Eligible individuals were aged 42 to 52 years, had an intact uterus and 1 or more ovaries, were not taking hormone therapy, had experienced 1 or more menses in the prior 3 months, and were members of the eligible ethnic/racial groups (ie, Black, Chinese, Hispanic, Japanese, and White). Follow-up in SWAN started in 1998, and follow-up visit 15 (ie, last cognitive testing) finished in 2017. Median (interquartile range) time between follow-up visits was 15.2 (1.4) years. In SWAN, cognitive performance was first used at follow-up 4; tests were repeated at follow-up visits 6 through 10 and follow-up visits 12, 13, and 15. The group with cognition measures consisted of 2709 participants. To minimize practice effects, this analysis’ observation period started at follow-up visit 7, which was the third testing occasion.^19,20,21^ Analysis sample requirements were cognitive measures at each of the first 3 cognitive visits (ie, follow-up visits 4, 6 and 7) and 1 or more cognitive measurements after analysis baseline (ie, follow-up visit 7). Stroke prior to baseline was an exclusion; if a stroke occurred after baseline, observations were censored. The Figure summarizes the sample derivation.

**Figure.**
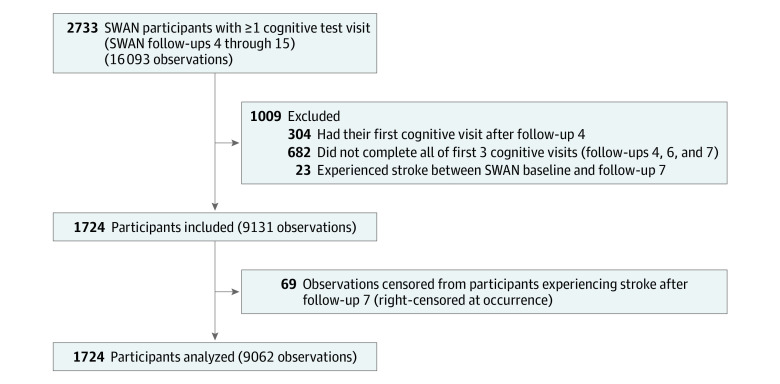
Derivation of the Analysis Sample SWAN indicates the Study of Women’s Health Across the Nation.

### Measurements

#### Outcomes

The Symbol Digit Modalities Test (SDMT; score range, 0-110) primarily assesses cognitive processing speed and complex attention.^22,23^ The SDMT also requires motor speed, visuospatial function, associative learning, and executive function; its broadness is associated with high discriminant validity and sensitivity to change.^22,24^ The East Boston Memory Test (EBMT; score range, 0-12) measures verbal episodic memory.^25^ The EBMT-immediate recall did not decrease with age in this or prior SWAN analyses; therefore, only the EBMT-delayed recall (EBMT-D) was included as an outcome. Working memory was assessed using the digit span backwards test (DSB, range 0-12).^26^ Scores in the SDMT, EBMT-D, and DSB each decrease in midlife, demonstrating sensitivity to aging-associated decline.^21,22,27,28^ Tests were professionally translated to pertinent languages.

#### Exposures

The self-administered Kaiser Physical Activity Survey (KPAS) was used to measure PA in SWAN.^29,30,31^ This survey quantifies PA during the past year by domain, including frequency of *household and caregiving* activities; frequency, duration, and perceived physical exertion of *sport and exercise* activities; and frequency of walking or biking for transportation and hours of television viewing, which is the *daily living* domain. Domain values range from 1 (lowest) to 5 (highest); their sum is total PA (range, 3-15). The KPAS is validated against activity logs, accelerometers, and maximal oxygen consumption levels; its indices, alone or in combination, explain 12% to 53% of the variance in these activity measures.^31^ The KPAS 1-month retest reliability (ie, intraclass correlation) ranges from 0.79 (for housework) to 0.84 (for sport and exercise activity).^31^ Because the vast majority of investigations of PA and cognition studied sport and exercise, that was our primary exposure.^4,17^ We conducted a secondary analysis using total PA. The KPAS was administered in SWAN at cohort baseline and follow-up visits 3, 5, 6, 9, 12, 13, and 15. Because SWAN did not assess PA at follow-up visits 7, 8, or 10, we imputed PA by computing the mean score for the visits preceding and following the unmeasured visit. At follow-up visits 11 and 14, SWAN did not test cognition; therefore, PA imputation was not required for these follow-up visits.

#### Covariates

Time-invariant characteristics were age at baseline, race/ethnicity, test-taking language; education (ie, ≤high school, some college, baccalaureate, or postgraduate education), site, and number of missed cognition assessments (an attrition estimate).^21,32^ Remaining factors could vary over time; for these, we assessed historical exposure prior to analysis baseline and time-varying exposure. Covariates were grouped into sociodemographic characteristics, menopause-associated variables, and comorbidities. The sole changeable sociodemographic factor was financial hardship (ie, very difficult or somewhat difficult to pay for basics, yes or no). Menopause-associated covariates included menopause symptoms, HT use, and menopause transition (MT) stage (ie, premenopausal, early perimenopausal, late perimenopausal, postmenopausal, or indeterminate).^8^ Use of systemic HT at each visit was categorized (ie, yes or no). Menopause symptoms included depressive, anxiety, sleep, and vasomotor symptoms.^20^ Comorbidities included diabetes (ie, diagnosis or using medication) and hypertension (ie, diagnosis, systolic blood pressure ≥130 mm Hg, diastolic blood pressure ≥85 mm Hg, or taking antihypertensive medications). We obtained height and weight using standard protocols.

### Statistical Analysis

We examined locally estimated scatterplot smoothing (LOESS) plots of cognition scores as a function of age at time of testing, starting at age 52 years, which was the mean age at baseline. Based on the functional form of the age-associated declines seen in the plots, we fit piecewise linear growth curves to repeated measurements of SDMT, DSB, and EBMT-D. Growth curves were parameterized by intercept (ie, baseline value) and slope (ie, rate of change over time during follow-up), allowing for a change of slope (ie, knot) when the woman reached a specific age. We used linear mixed effects regression with random intercept and random slopes (before and after fixed knots, as described subsequently) at the participant level. We tested for the presence of a practice effect from the third cognitive test (ie, the analysis baseline) to the fourth test as a fixed offset from the third test to the fourth and later tests, and for an additional practice effect from the fourth to fifth test; for each, we included a fixed and random effect. We tested appropriateness of knot locations using a previously published method.^32^ For SDMT and DSB, optimal knot location was age 61 years; for EBMT-D, it was age 58 years. SDMT demonstrated a significant practice effect from the third to fourth visit. No evidence of a practice effect was found with DSB or EBMT-D.

In the base model (ie, model 1), time-varying PA was added to the null model (which included attrition and practice effect). The PA level at the time of the cognition testing was allowed to be associated with the outcome of contemporary cognition score, and the mean of PA at 2 successive visits was allowed to be associated with the outcome of cognition slope between the 2 visits. Declines in SDMT were statistically different from zero before and after the knot at age 61 years, but decreases in EBMT-D score were evident after the knot at age 58 years, and decreases in DSB scores were evident after the knot at age 61 years. Therefore, an association between PA and slope was modeled only at and after age 58 years for EBMT-D and age 61 years for DSB. We used a staged, nested modeling approach. Model 1 included the sport and exercise or total PA exposure and practice effect (SDMT only); it modeled cognition scores to be different by the number of cognitive assessments missed (ie, attrition). Model 2 added sociodemographic characteristics (ie, age, race/ethnicity, language, education, study site, and difficulty paying for basic necessities) and menopause-associated factors (ie, menopause transition stage, hormone therapy use, and symptoms) and allowed the rate of change of cognition score to be different by number of cognitive assessments missed (ie, attrition).^33,34,35^ Model 3 additionally controlled for the presence or absence of diabetes and hypertension.

Historical exposure to time-varying characteristics was captured as percent of prior visits (ie, SWAN baseline through follow-up visit 6) at which the characteristic was present or, in the case of PA, the mean of prior scores. Historical exposures were allowed to be associated with starting level of the outcome. Time-invariant and time-varying covariates at analysis baseline and later were permitted to be associated with the outcome of contemporaneous cognitive levels; time-invariant characteristics and starting values of time-varying covariates were allowed to be associated with the outcome of slope of cognitive change. All covariates were modeled as having the same association with SDMT slope prior to or after age 61 years. Covariates were modeled as being associated with slopes after knots at age 58 years for EBMT-D and 61 years for DSB.

Based on the results of models using the sport and exercise PA exposure, cognitive test trajectories were graphed for 5 groups of women. The 5 groups were the referent participant, women who were more physically active than the referent, women who were less physically active than the referent, women with some financial strain, and women with severe financial strain.

All analyses were repeated using total PA level as the primary exposure. *P* values were 2-sided, and statistical significance was set at *P* ≤ .05. Data analysis was performed from June 2018 through August 2019 using SAS statistical software version 9.4 (SAS Institute).

## Results

Among 1718 women in the analytic sample, 458 women were Black (26.6%), 181 women were Chinese (10.5%), 210 women were Japanese (12.2%), and 869 women were White (50.6%). Mean (SD) age at baseline was 45.7 (2.5) years. There were 32 women (1.9%) who were premenopausal, 427 women (24.9%) who were early perimenopausal, 170 women (9.9%) who were late perimenopausal, and 947 women (55.1%) who were postmenopausal. In 142 women (8.3%), menopause transition stage was unclassifiable. Among participants in the analytic sample, 49 women (2.9%) did not have a high school degree, 246 women (14.3%) were high school graduates, 550 women (32.0%) attended some college, 395 women (23.0%) completed college, and 478 women (27.8)% had postbaccalaureate education. The test-taking language was English for 1573 women (91.6%), Chinese for 69 women (4.0%), and Japanese for 76 women (4.4%). We followed the sample for a median (range) of 11.9 (0.60-13.5) years.

At baseline, crude mean SDMT and DSB scores approximated their range midpoints, with symmetrical distributions (Table 1). Mean (SD) baseline crude EBMT-D score was 10.3 (1.7); 573 women (33.4%) began with the maximum score.^13^ Mean (SD) sport and exercise PA score was 2.79 (0.96) at baseline. At baseline, mean (SD) total PA was 7.59 (1.64),

**Table 1.** Crude Values for Outcomes and Covariates at Baseline and Follow-Up Visits

	SWAN,^a^^,^^b^ mean (SD)			
	Visit 7 (n = 1528-1724)	Visits 8 and 9 (n = 1460-1604)	Visit 12 (n = 1438-1489)	Visit 15 (n = 1226-1270)
Outcome				
Symbol digit modalities test	58.19 (10.72)	58.94 (10.96)	57.58 (10.78)	55.70 (10.81)
Missing data, No. (%)	6 (0.35)	7 (0.44)	3 (0.20)	7 (0.55)
Digit span backwards	6.95 (2.32)	6.96 (2.32)	6.99 (2.30)	6.80 (2.25)
Missing data, No. (%)	43 (2.50)	33 (2.06)	18 (1.21)	35 (2.76)
East Boston Memory Test-Delayed	10.27 (1.73)	10.43 (1.65)	10.34 (1.68)	10.15 (1.68)
Missing data, No. (%)	2 (0.12)	1 (0.06)	1 (0.07)	1 (0.08)
Primary exposure				
Sport and exercise physical activity level	2.79 (0.96)	2.79 (0.99)	2.89 (1.03)	2.90 (1.03)
Missing data, No. (%)	195 (11.35)	144 (8.98)	16 (1.07)	30 (2.36)
Total physical activity level	7.59 (1.64)	7.55 (1.71)	7.66 (1.82)	7.57 (1.82)
Missing data, No. (%)	199 (11.58)	147 (9.16)	18 (1.21)	34 (2.68)
Covariate, No. (%)				
Financial hardship				
High level	73 (4.31)	59 (3.77)	71 (4.85)	22 (1.77)
Moderate level	347 (20.48)	307 (19.60)	309 (21.09)	186 (14.94)
Missing data, No. (%)	24 (1.40)	38 (2.37)	24 (1.61)	25 (1.97)
Hormone therapy use	278 (16.53)	180 (11.42)	68 (4.73)	33 (2.69)
Missing data, No. (%)	36 (2.10)	28 (1.75)	51 (3.43)	44 (3.46)
Depressive symptoms	345 (20.31)	324 (20.66)	264 (17.73)	201 (15.83)
Missing data, No. (%)	19 (1.11)	36 (2.24)	0	0
Sleep problems	482 (28.37)	446 (28.30)	474 (31.96)	455 (36.11)
Missing data, No. (%)	19 (1.11)	28 (1.75)	6 (0.40)	10 (0.79)
Vasomotor symptoms	454 (26.49)	428 (26.88)	256 (17.24)	140 (11.10)
Missing data, No. (%)	4 (0.23)	12 (0.75)	4 (0.27)	9 (0.71)
Anxiety symptoms	538 (31.39)	485 (30.46)	361 (24.36)	254 (20.17)
Missing data, No. (%)	4 (0.23)	12 (0.75)	7 (0.47)	11 (0.87)
Diabetes	116 (6.75)	140 (8.73)	186 (12.49)	189 (14.88)
Hypertension	676 (39.62)	648 (40.53)	765 (51.45)	730 (57.71)
Missing data, No. (%)	12 (0.70)	5 (0.31)	2 (0.13)	5 (0.39)
BMI	28.52 (7.09)	28.51 (7.06)	28.96 (7.22)	28.65 (6.86)
Missing data, No. (%)	15 (0.87)	15 (0.94)	15 (1.01)	9 (0.71)

Abbreviations: BMI, body mass index (calculated as weight in kilograms divided by height in meters squared); SWAN, Study of Women’s Health Across the Nation.

^a^ SWAN follow-up visit 7 is the baseline visit for the current analysis, and SWAN follow-up visit 15 is the final visit. Visits included in this table were selected to be approximately equally spaced across the study’s observation period. Due to budget constraints, approximately half of the SWAN cohort underwent cognitive tests at follow-up visit 8 and the remainder at follow-up visit 9.

^b^ Range in sample sizes are due to missing values for some variables.

In models that included practice effect and attrition as covariates, from baseline through age 61 years, the yearly rate of change in SDMT score was −0.21 (95% CI, −0.26 to −0.15; *P* < .001). After age 61 years, the yearly rate of change in mean SDMT score was −0.51 annually (95% CI, −0.54 to −0.41; *P* < .001). We found a statistically significant practice effect for mean SDMT score, which increased by 0.6 units from baseline to later tests (95% CI, 0.2 to 0.9; *P* = .001). Each missed visit was associated with a decrement of 1.30 from initial mean SDMT score (95% CI, 0.88 to 1.72; *P* < .001). In contrast, mean EBMT-D score did not change significantly between baseline and age 58 years, and mean DSB score did not change significantly between baseline and age 61 years. Statistically significant decreases were found at later ages. Mean annual change in EBMT-D score was −0.03 yearly (95% CI, −0.04 to −0.02; *P* < .001) after age 58 years. Mean annual change in DSB score was −0.03 (95% CI, −0.04 to −0.01; *P* = .001) after age 61 years. For each missed test, baseline EBMT-D score was 0.13 lower (95% CI, 0.08 to 0.19; *P* < .001) and baseline mean DSB was 0.16 lower (95% CI, 0.08 to 0.25; *P* < .001)

In model 1, which was adjusted for practice effect and attrition, sport and exercise PA level was positively associated with concurrent SDMT level and annual slope (Table 2). Every unit increment in sport or exercise PA level was associated with a 0.36 increment in concurrent SDMT score (95% CI, 0.14 to 0.59; *P* = .002) and 0.06 smaller annual decline in SDMT score (95% CI, 0.02 to 0.09; *P* = .001). After adjustment for demographic characteristics and menopause-associated factors, associations between sport and exercise PA levels and SDMT level and slope decreased in magnitude and were not statistically significant (Table 2). Race/ethnicity and education were statistically significantly associated with baseline differences in SDMT score. Higher values of past anxiety, past depression, and current depression were significantly associated with lower SDMT starting values. Scores for SDMT were also statistically significantly lower in late perimenopause compared with postmenopause. Among the demographic characteristics and menopause-associated covariates, financial hardship and concurrent anxiety level were associated with greater declines in SDMT score. For each unit increment in the anxiety scale score, the rate of change in in SDMT score was −0.09 (95% CI, −0.16 to −0.01; *P* = .02). The associations found in Model 2 persisted after adjustment for diabetes and hypertension (Table 2). Neither hypertension nor diabetes was statistically significantly associated with SDMT change rate.

**Table 2.** Adjusted Association of Sport and Exercise Physical Activity With Symbol Digits Modalities Test Score Trajectories

	Model 1^a^				Model 2^b^					Model 3^c^			
	SDMT level (95% CI)	*P* value	SDMT annual slope (95% CI)	*P* value	SDMT level (95% CI)		*P* value	SDMT annual slope (95% CI)	*P* value	SDMT level (95% CI)	*P* value	SDMT annual slope (95% CI)	*P* value
SDMT value in referent woman^d^	Starting level: 58.11 (57.23 to 58.99)	<.001	Before age 61 y: −0.21 (−0.26 to −0.15)	<.001	Starting level: 60.72 (59.00 to 62.45)	<.001		Before age 61 y: −0.2 (−0.39 to −0.12)5	<.001	Starting level: 60.88 (59.13 to 62.63)	<.001	Before age 61 y: −0.24 (−0.37 to −0.1)	<.001
			After age 61 y: −0.51 (−0.57 to −0.44)	<.001				After age 61 y: −0.47 (−0.62 to −0.33)	<.001			After age 61 y: −0.46 (−0.61 to −0.31)	<.001
Variable^e^													
Sport and exercise PA level	0.36 (0.14 to 0.59)	.002	0.06 (0.02 to 0.09)	.001	0.12 (−0.11 to 0.35)	.30		0.02 (−0.02 to 0.06)	.26	0.11 (−0.12 to 0.35)	.35	0.02 (−0.02 to 0.05)	.38
No. missed visits	−1.12 (−1.60 to −0.64)	<.001	NA	NA	−0.67 (−1.12 to −0.22)	.004		−0.11 (−0.17 to −0.04)	.002	−0.62 (−1.08 to −0.17)	.007	−0.10 (−0.17 to −0.04)	.002
Demographic characteristic													
Race/ethnicity													
Chinese	NA	NA	NA	NA	−1.24 (−3.49 to 1.00)	.28		0.02 (−0.14 to 0.17)	.82	−1.21 (−3.45 to 1.03)	.29	0.01 (−0.14 to 0.17)	.87
Japanese	NA	NA	NA	NA	1.14 (−0.98 to 3.25)	.29		0.06 (−0.09 to 0.21)	.42	1.22 (−0.9 to 3.34)	.26	0.07 (−0.08 to 0.22)	.36
Black	NA	NA	NA	NA	−5.87 (−7.15 to −4.59)	<.001		0 (−0.1 to 0.09)	.93	−5.64 (−6.96 to −4.31)	<.001	0 (−0.1 to 0.09)	.98
Education		NA		NA									
High school or less	NA	NA	NA	NA	−2.90 (−4.28 to −1.51)	<.001		0.04 (−0.06 to 0.14)	.47	−2.66 (−4.05 to −1.27)	<.001	0.03 (−0.07 to 0.13)	.60
College or some college	NA	NA	NA	NA	1.79 (0.53 to 3.05)	.005		0.02 (−0.07 to 0.11)	.66	1.76 (0.5 to 3.03)	.006	0.02 (−0.07 to 0.11)	.63
Postbaccalaureate	NA	NA	NA	NA	2.20 (0.95 to 3.45)	<.001		−0.03 (−0.12 to 0.06)	.51	2.13 (0.88 to 3.38)	<.001	−0.02 (−0.11 to 0.06)	.57
Testing language		NA		NA									
Chinese	NA	NA	NA	NA	−0.74 (−2.96 to 1.49)	.52		−0.14 (−0.34 to 0.06)	.16	−0.83 (−3.05 to 1.4)	.47	−0.13 (−0.33 to 0.07)	.19
Japanese	NA	NA	NA	NA	2.53 (0.005 to 5.05)	.0496		−0.01 (−0.19 to 0.17)	.91	2.37 (−0.15 to 4.89)	.07	−0.01 (−0.19 to 0.17)	.92
Economic hardship		NA		NA									
Somewhat hard	NA	NA	NA	NA	−0.24 (−0.71 to 0.23)	.31		−0.12 (−0.2 to −0.03)	.008	−0.20 (−0.67 to 0.28)	.41	−0.12 (−0.21 to −0.03)	.007
Very hard	NA	NA	NA	NA	−0.29 (−1.24 to 0.65)	.54		−0.24 (−0.42 to −0.06)	.01	−0.24 (−1.19 to 0.71)	.62	−0.23 (−0.41 to −0.04)	.02
**Menopause-associated variable**													
Hormone therapy use		NA		NA									
Past	NA	NA	NA	NA	−0.63 (−2.48 to 1.22)	.51		NA	NA	−0.42 (−2.28 to 1.43)	.66	NA	NA
Current	NA	NA	NA	NA	0.17 (−0.55 to 0.9)	.64		−0.04 (−0.14 to 0.06)	.47	0.20 (−0.52 to 0.93)	.58	−0.04 (−0.14 to 0.06)	.48
Depressive symptoms		NA		NA									
Past	NA	NA	NA	NA	−3.18 (−5.16 to −1.19)	.002		NA	NA	−3.23 (−5.22 to −1.24)	.002	NA	NA
Current	NA	NA	NA	NA	−0.49 (−0.94 to −0.04)	.03		−0.01 (−0.1 to 0.08)	.80	−0.45 (−0.90 to 0.005)	.052	−0.01 (−0.1 to 0.08)	.84
Anxiety symptoms		NA		NA									
Past	NA	NA	NA	NA	1.25 (−0.6 to 3.1)	.19		NA	NA	1.35 (−0.50 to 3.21)	.15	NA	NA
Current	NA	NA	NA	NA	−0.62 (−1.03 to −0.21)	.003		−0.08 (−0.16 to −0.01)	.03	−0.61 (−1.02 to −0.20)	.004	−0.09 (−0.16 to −0.01)	.02
Sleep problems		NA		NA									
Past	NA	NA	NA	NA	0.12 (−1.58 to 1.82)	.89		NA	NA	0.16 (−1.54 to 1.86)	.86	NA	NA
Current	NA	NA	NA	NA	0.11 (−0.26 to 0.47)	.57		0.02 (−0.05 to 0.09)	.60	0.14 (−0.23 to 0.51)	.46	0.02 (−0.05 to 0.09)	.61
Vasomotor symptoms		NA		NA									
Past	NA	NA	NA	NA	−0.12 (−2.18 to 1.94)	.91		NA	NA	−0.12 (−2.18 to 1.95)	.91	NA	NA
Current		NA		NA	−0.18 (−0.59 to 0.23)	.38		0.01 (−0.06 to 0.09)	.71	−0.18 (−0.59 to 0.23)	.39	0.01 (−0.06 to 0.09)	.70
Perimenopause		NA		NA									
Pre or early	NA	NA	NA	NA	0.37 (−0.37 to 1.10)	.33		0.02 (−0.07 to 0.12)	.62	0.36 (−0.38 to 1.09)	.34	0.03 (−0.07 to 0.13)	.56
Late	NA	NA	NA	NA	−0.78 (−1.50 to −0.06)	.03		0.05 (−0.06 to 0.17)	.37	−0.77 (−1.49 to −0.05)	.04	0.05 (−0.06 to 0.17)	.38
**Cardiometabolic risk factor**													
Hypertension		NA		NA									
Past	NA	NA	NA	NA	NA	NA		NA	NA	−0.23 (−1.6 to 1.14)	.74	NA	NA
Current	NA	NA	NA	NA	NA	NA		NA	NA	−0.41 (−0.87 to 0.04)	.08	−0.03 (−0.11 to 0.04)	.38
Diabetes		NA		NA									
Past	NA	NA	NA	NA	NA	NA		NA	NA	−2.68 (−5.64 to 0.27)	.08	NA	NA
Current	NA	NA	NA	NA	NA	NA		NA	NA	−0.28 (−1.08 to 0.53)	.496	0.07 (−0.07 to 0.22)	.31

Abbreviations: NA, not applicable; PA, physical activity; SDMT, Symbol Digit Modalities Test.

^a^ Model 1 (base model) covariates: No. of missed visits, practice effect (a fixed increment in score from analysis baseline to all following visits), and random effects for intercept, 2 slopes, and the practice effect. The maximum No. of women with SDMT values in the analysis sample was 1718. Owing to missing values for 1 or more covariates, the sample size for model 1 was 1523.

^b^ Model 2 covariates: variables in model 1, Study of Women’s Health Across the Nation study site, and each of the demographic characteristics and menopause-associated factors listed in the table. Economic hardship was operationalized as difficulty paying for basic necessities, such as food. The maximum number of women with SDMT values in the analysis sample was 1718. Owing to missing values for 1 or more covariates, the sample size for model 2 was 1472.

^c^ Model 3 covariates: variables in model 2 and cardiometabolic risk factors listed in the table. The maximum number of women with SDMT values in the analysis sample was 1718. Owing to missing values for 1 or more covariates, the sample size for model 3 was 1464.

^d^ The referent woman participated in all Study of Women’s Health Across the Nation cognition visits and had a sport and exercise PA score of 2.77 (scale, 1-5). For models 2 and 3, the referent woman was age 54 years at analysis baseline; was White; had a high school education; took tests in English; reported no financial hardship; did not use hormone therapy; did not report high levels (ie, top quartile) of depressive symptoms, anxiety symptoms, or vasomotor symptoms; and was postmenopausal at analysis baseline. For model 3, the referent woman did not report hypertension or diabetes at any visit.

^e^ For time-varying covariates, past exposure was assessed between Study of Women’s Health Across the Nation cohort baseline and current study analysis baseline (ie, Study of Women’s Health Across the Nation follow-up visit 7). Past exposure was operationalized as the proportion (range, 0 to 1) of visits prior to analysis baseline that the participant reported the exposure. Current values (ie, starting at analysis baseline) of time-varying covariates were modeled to be associated with the outcome of SDMT score level at the same visit (ie, contemporaneously), and the value at study baseline was modeled to be associated with the outcome of SDMT slope. However, the PA level at each visit was modeled as being associated with the outcome of slope only up to the next visit.

In model 1, sport and exercise PA level was positively associated with current EBMT-D score but not with decrease in EBMT-D score (Table 3). Each unit increment in sport and exercise PA level was associated with a 0.10 increment in EBMT-D score (95% CI, 0.05-0.15; *P* < .001). There was not a statistically significant association after adjusting for demographic characteristics and menopause-related factors (ie, model 2). In this model, statistically significant differences were found in EBMT-D level by race/ethnicity, educational attainment, and current financial hardship. Histories of depression and vasomotor symptoms were associated with smaller baseline EBMT-D scores. However, socioeconomic-associated factors and menopause-associated factors were not associated with the rate of decrease in EBMT-D after age 58 years. All significant associations with EBMT-D score found in model 2 persisted after additional adjustment for hypertension and diabetes (ie, model 3). Neither hypertension nor diabetes was statistically significantly associated with the level or annual slope of EBMT-D score.

**Table 3.** Adjusted Associations of Sport and Exercise Physical Activity With East Boston Memory Test-Delayed Score Trajectories

	Model 1^a^				Model 2^b^				Model 3^c^			
	EBMT-D level (95% CI)	*P* value	EBMT-D annual slope (95% CI)	*P* value	EBMT-D level (95% CI)	*P* value	EBMT-D annual slope (95% CI)	*P* value	EBMT-D level (95% CI)	*P* value	EBMT-D annual slope (95% CI)	*P* value
EBMT-D value in referent woman^d^	Starting level: 10.14 (9.98 to 10.29)	<.001	Before age 58 y: 0.01 (−0.01 to 0.02)	.46	Starting level: 10.72 (10.47 to 10.97)	<.001	Before age 58 y: 0.01 (−0.01 to 0.03)	.38	Starting level: 10.70 (10.45 to 10.96)	<.001	Before age 58 y: 0.01 (−0.01 to 0.03)	.47
			After age 58 y: −0.03 (−0.05 to −0.02)	<.001			After age 58 y: −0.08 (−0.12 to −0.04)	<.001			After age 58 y: −0.08 (−0.12 to −0.04)	<.001
Variable^e^												
Sport and exercise PA level	0.10 (0.05 to 0.15)	<.001	0 (−0.01 to 0.01)	.81	0.04 (−0.01 to 0.09)	.14	−0.01 (−0.02 to 0.002)	.12	0.04 (−0.01 to 0.09)	.13	−0.01 (−0.02 to 0.003)	.12
No. missed visits	−0.12 (−0.18 to −0.06)	<.001	NA	NA	−0.05 (−0.11 to 0.01)	.12	−0.02 (−0.05 to 0.002)	.07	−0.05 (−0.12 to 0.01)	.09	−0.02 (−0.05 to 0.003)	.09
Demographic characteristic												
Race/ethnicity												
Chinese	NA	NA	NA	NA	−0.51 (−0.8 to −0.21)	<.001	−0.06 (−0.12 to −0.01)	.02	−0.50 (−0.8 to −0.21)	<.001	−0.06 (−0.12 to −0.01)	.02
Japanese	NA	NA	NA	NA	−0.31 (−0.59 to −0.03)	.03	−0.01 (−0.07 to 0.04)	.68	−0.32 (−0.59 to −0.04)	.03	−0.01 (−0.06 to 0.05)	.76
Black	NA	NA	NA	NA	−0.48 (−0.65 to −0.32)	<.001	−0.02 (−0.05 to 0.01)	.25	−0.50 (−0.67 to −0.33)	<.001	−0.02 (−0.05 to 0.02)	.35
Education												
High school or less	NA	NA	NA	NA	−0.32 (−0.5 to −0.14)	<.001	−0.01 (−0.04 to 0.03)	.74	−0.31 (−0.49 to −0.13)	<.001	0 (−0.04 to 0.03)	.86
College or some college	NA	NA	NA	NA	0.27 (0.09 to 0.42)	.002	−0.01 (−0.04 to 0.03)	.71	0.27 (0.1 to 0.43)	.001	0 (−0.04 to 0.03)	.79
Postbaccalaureate	NA	NA	NA	NA	0.21 (0.05 to 0.37)	.01	0 (−0.03 to 0.03)	.97	0.22 (0.06 to 0.38)	.008	0 (−0.03 to 0.03)	.98
Testing language												
Chinese	NA	NA	NA	NA	−0.36 (−0.72 to −0.002)	.048	0.03 (−0.04 to 0.11)	.39	−0.36 (−0.72 to −0.002)	.049	0.03 (−0.04 to 0.1)	.40
Japanese	NA	NA	NA	NA	−0.21 (−0.55 to 0.14)	.24	−0.05 (−0.12 to 0.02)	.14	−0.20 (−0.54 to 0.15)	.26	−0.05 (−0.12 to 0.01)	.13
Economic hardship												
Somewhat hard	NA	NA	NA	NA	−0.02 (−0.13 to 0.09)	.71	0.01 (−0.02 to 0.04)	.42	−0.02 (−0.12 to 0.09)	.77	0.01 (−0.02 to 0.04)	.42
Very hard	NA	NA	NA	NA	−0.29 (−0.5 to −0.08)	.01	−0.05 (−0.12 to 0.01)	.12	−0.29 (−0.51 to −0.08)	.007	−0.05 (−0.12 to 0.02)	.17
**Menopause-associated variable**													
Hormone therapy												
Past	NA	NA	NA	NA	0.13 (−0.1 to 0.37)	.27	NA	NA	0.14 (−0.1 to 0.38)	.25	NA	NA
Current	NA	NA	NA	NA	−0.01 (−0.16 to 0.15)	.92	0.02 (−0.01 to 0.05)	0.26	0.01 (−0.16 to 0.15)	.92	0.02 (−0.01 to 0.05)	.23
Depressive symptoms												
Past	NA	NA	NA	NA	−0.30 (−0.56 to −0.05)	.02	NA	NA	−0.31 (−0.56 to −0.06)	.02	NA	NA
Current	NA	NA	NA	NA	−0.01 (−0.11 to 0.1)	.90	0.01 (−0.02 to 0.04)	.69	0 (−0.11 to 0.11)	.97	0.01 (−0.02 to 0.04)	.64
Anxiety symptoms												
Past	NA	NA	NA	NA	−0.01 (−0.25 to 0.22)	.92	NA	NA	−0.02 (−0.25 to 0.22)	.88	NA	NA
Current	NA	NA	NA	NA	0.02 (−0.08 to 0.11)	.75	0 (−0.03 to 0.02)	.90	0.01	.82	0 (−0.03 to 0.03)	.98
Sleep problems												
Past	NA	NA	NA	NA	0.03 (−0.18 to 0.25)	.76	NA	NA	0.03 (−0.19 to 0.24)	.82	NA	NA
Current	NA	NA	NA	NA	−0.07 (−0.16 to 0.02)	.13	−0.01 (−0.03 to 0.02)	.64	−0.07 (−0.16 to 0.02)	.11	0 (−0.03 to 0.02)	.79
Vasomotor symptoms												
Past	NA	NA	NA	NA	−0.32 (−0.58 to −0.06)	.01	NA	NA	−0.32 (−0.58 to −0.06)	.02	NA	NA
Current		NA		NA	0.08 (−0.01 to 0.18)	.08	0.01 (−0.01 to 0.04)	.26	0.10 (0.002 to 0.19)	.045	0.01 (−0.01 to 0.04)	.29
Perimenopause												
Pre or early	NA	NA	NA	NA	0 (−0.15 to 0.15)	.98	0 (−0.04 to 0.03)	.83	−0.01 (−0.16 to 0.15)	.95	0 (−0.04 to 0.03)	.81
Late	NA	NA	NA	NA	−0.06 (−0.23 to 0.11)	.48	−0.01 (−0.05 to 0.03)	.74	−0.06 (−0.23 to 0.1)	.45	−0.01 (−0.05 to 0.03)	.63
**Cardiometabolic risk factors**												
Hypertension												
Past	NA	NA	NA	NA	NA	NA	NA	NA	0.07 (−0.12 to 0.26)	.47	NA	NA
Current	NA	NA	NA	NA	NA	NA	NA	NA	−0.01 (−0.12 to 0.09)	.82	−0.01 (−0.04 to 0.01)	.41
Diabetes												
Past	NA	NA	NA	NA	NA	NA	NA	NA	−0.16 (−0.56 to 0.24)	.43	NA	NA
Current	NA	NA	NA	NA	NA	NA	NA	NA	0.09 (−0.08 to 0.26)	.31	−0.02 (−0.08 to 0.03)	.32

Abbreviations: EBMT-D, East Boston Memory Test-Delayed; NA, not applicable; PA, physical activity.

^a^ Model 1 covariates: No. of missed visits, practice effect (a fixed increase in score from analysis baseline to all following visits), and random associations for intercept and 2 slopes. The maximum No. of women with EBMT-D values in the analysis sample was 1718. Owing to missing values for 1 or more covariates, the sample size for model 1 was 1580.

^b^ Model 2 covariates: variables in model 1, Study of Women’s Health Across the Nation study site and each of the demographic characteristics and menopause-associated factors listed in the table. Economic hardship was operationalized as difficulty paying for basic necessities, such as food. The maximum No. of women with EBMT-D values in the analysis sample was 1718. Owing to missing values for 1 or more covariates, the sample size for model 2 was 1523.

^c^ Model 3 covariates: variables in model 2 and cardiometabolic risk factors listed in the table. The maximum number of women with EBMT-D values in the analysis sample was 1718. Owing to missing values for 1 or more covariates, the sample size for model 3 was 1514.

^d^ The referent woman participated in all Study of Women’s Health Across the Nation cognition visits and had a sport and exercise PA score of 2.77 (scale, 1-5). For models 2 and 3, the referent woman was age 54 years at analysis baseline; was White; had a high school education; took tests in English; reported no financial hardship; did not use hormone therapy; did not report high levels (ie, top quartile) of depressive symptoms, anxiety symptoms, or vasomotor symptoms; and was postmenopausal at analysis baseline. For model 3, the referent woman did not report hypertension or diabetes at any visit.

^e^ For time-varying covariates, past exposure was assessed between Study of Women’s Health Across the Nation cohort baseline and current study analysis baseline (ie, Study of Women’s Health Across the Nation follow-up visit 7). Past exposure was operationalized as the proportion (range, 0 to 1) of visits prior to analysis baseline that the participant reported the exposure. Current values (ie, starting at analysis baseline) of time-varying covariates were modeled to be associated with the outcome of EBMT-D score level at the same visit (ie, contemporaneously), and the value at study baseline was modeled to be associated with the outcome of EBMT-D slope. However, PA level at each visit was modeled as being associated with the outcome of slope only up to the next visit.

In model 1, sport and exercise PA level was not associated with DSB score or annual rate of decline in DSB score (Table 4). In model 2, statistically significant differences in DSB score by race/ethnicity and graded associations of DSB with education level were found. Current anxiety and sleep problems were associated with a lower DSB score. Although financial hardship was not associated with DSB level, it was associated with a faster decrease in DSB. After controlling for demographic characteristics and menopause-associated variables (ie, model 2), there was no association between age and decrease in DSB score; DSB slope after age 61 years was −0.03 per year (95% CI, −0.09 to 0.39; *P* = .44). After additional adjustment for hypertension and diabetes (ie, model 3), significant associations of demographics and menopause-associated factors with DSB levels and annual rates of change persisted, but these demographic characteristics and menopause-associated factors were not associated with DSB slope.

**Table 4.** Adjusted Associations of Sport and Exercise Physical Activity With Digit Span Backwards Score Trajectories

	Model 1^a^				Model 2^b^				Model 3^c^			
	DSB level (95% CI)	*P* value	DSB annual slope (95% CI)	*P* value	DSB level (95% CI)	*P* value	DSB annual slope (95% CI)	*P* value	DSB level (95% CI)	*P* value	DSB annual slope (95% CI)	*P* value
DSB value in referent woman^d^	Starting level: 6.91 (6.71 to 7.11)	<.001	Before age 61 y: 0 (−0.01 to 0.01)	.89	Starting level: 7.21 (6.85 to 7.58)	<.001	Before age 61 y: 0 (−0.01 to 0.02)	.78	Starting level: 7.23 (6.86 to 7.60)	<.001	Before age 61 y: 0 (−0.01 to 0.02)	.63
			After age 61 y: −0.03 (−0.05 to −0.01)	.003			After age 61 y: −0.03 (−0.09 to 0.04)	.44			After age 61 y: 0.02 (−0.09 to 0.04)	.502
Variable^e^												
Sport and exercise PA level	0.03 (−0.02 to 0.09)	.23	0.01 (−0.01 to 0.02)	.21	−0.03 (−0.09 to 0.02)	.25	0 (−0.01 to 0.02)	.73	−0.03 (−0.09 to 0.02)	.23	0 (−0.01 to 0.02)	.57
No. missed visits	−0.11 (−0.20 to 0.01)	.03	NA	NA	−0.08 (−0.17 to 0.02)	.11	−0.03 (−0.07 to 0.01)	.12	−0.07 (−0.17 to 0.02)	.14	−0.03 (−0.07 to 0.01)	.14
Demographic												
Race/ethnicity												
Chinese	NA	NA	NA	NA	−0.79 (−1.25 to −0.33)	<.001	−0.02 (−0.11 to 0.06)	.61	−0.79 (−1.25 to −0.33)	<.001	−0.02 (−0.11 to 0.06)	.60
Japanese	NA	NA	NA	NA	−0.48 (−0.92 to −0.05)	.03	0.03 (−0.05 to 0.11)	.47	−0.48 (−0.92 to −0.04)	.03	0.03 (−0.05 to 0.12)	.48
Black	NA	NA	NA	NA	−1.31 (−1.57 to −1.06)	<.001	0.02 (−0.03 to 0.07)	.42	−1.26 (−1.52 to −1.00)	<.001	0.02 (−0.04 to 0.07)	.55
Education												
High school or less	NA	NA	NA	NA	−0.47 (−0.75 to −0.20)	<.001	−0.01 (−0.06 to 0.05)	.84	−0.46 (−0.74 to −0.17)	.002	−0.01 (−0.06 to 0.04)	.73
College or some college	NA	NA	NA	NA	0.48 (0.23 to 0.74)	<.001	0.01 (−0.04 to 0.07)	.58	0.48 (0.23 to 0.74)	<.001	0.01 (−0.04 to 0.06)	.63
Postbaccalaureate	NA	NA	NA	NA	0.85 (0.60 to 1.10)	<.001	−0.02 (−0.07 to 0.03)	.37	0.85 (0.60 to 1.10)	<.001	−0.02 (−0.07 to 0.02)	.34
Test language												
Chinese	NA	NA	NA	NA	1.28 (0.81 to 1.76)	<.001	0.05 (−0.06 to 0.16)	.39	1.28 (0.81 to 1.76)	<.001	0.05 (−0.06 to 0.16)	.35
Japanese	NA	NA	NA	NA	0.48 (−0.05 to 1.00)	.07	−0.08 (−0.18 to 0.02)	.11	0.47 (−0.06 to 0.99)	.08	−0.07 (−0.17 to 0.02)	.14
Economic hardship		NA		NA								
Somewhat hard	NA	NA	NA	NA	−0.08 (−0.20 to 0.04)	.19	−0.06 (−0.11 to −0.01)	.01	−0.08 (−0.19 to 0.04)	.21	−0.07 (−0.12 to −0.02)	.01
Very hard	NA	NA	NA	NA	−0.02 (−0.26 to 0.21)	.84	−0.03 (−0.16 to 0.09)	.63	−0.03 (−0.26 to 0.21)	.81	−0.05 (−0.18 to 0.07)	.42
**Menopause-associated variable**												
Hormone therapy												
Past	NA	NA	NA	NA	−0.37 (−0.75 to 0.003)	.05	NA	NA	−0.36 (−0.73 to 0.02)	.07	NA	NA
Current	NA	NA	NA	NA	0.17 (−0.003 to 0.35)	.05	0.03 (−0.02 to 0.08)	.30	0.17 (−0.003 to 0.35)	.054	0.03 (−0.02 to 0.08)	.32
Depressive symptoms												
Past	NA	NA	NA	NA	−0.28 (−0.69 to 0.12)	.17	NA	NA	−0.31 (−0.72 to 0.10)	.14	NA	NA
Current	NA	NA	NA	NA	0.07 (−0.04 to 0.18)	.23	0 (−0.05 to 0.05)	.95	0.07 (−0.04 to 0.19)	.21	0 (−0.05 to 0.05)	.89
Anxiety symptoms												
Past	NA	NA	NA	NA	0.10 (−0.28 to 0.48)	.60	NA	NA	0.11 (−0.26 to 0.50)	.55	NA	NA
Current	NA	NA	NA	NA	−0.10 (−0.21 to −0.001)	.048	−0.03 (−0.07 to 0.02)	.21	−0.10 (−0.21 to −0.002)	.045	−0.03 (−0.07 to 0.01)	.20
Sleep problems												
Past	NA	NA	NA	NA	0.21 (−0.14 to 0.56)	.25	NA	NA	0.20 (−0.15 to 0.55)	.26	NA	NA
Current	NA	NA	NA	NA	−0.13 (−0.22 to −0.04)	.007	0 (−0.04 to 0.04)	.93	−0.13 (−0.22 to −0.04)	.006	0 (−0.05 to 0.04)	.82
Vasomotor symptoms												
Past	NA	NA	NA	NA	−0.19 (−0.62 to 0.23)	.38	NA	NA	−0.16 (−0.59 to 0.27)	.47	NA	NA
Current	NA	NA	NA	NA	0.07 (−0.04 to 0.17)	.21	−0.01 (−0.05 to 0.04)	.75	0.07 (−0.03 to 0.17)	.18	−0.01 (−0.05 to 0.04)	.77
Perimenopause												
Pre or early	NA	NA	NA	NA	−0.02 (−0.19 to 0.14)	.77	0 (−0.06 to 0.07)	.89	−0.03 (−0.19 to 0.14)	.76	−0.01 (−0.07 to 0.06)	.88
Late	NA	NA	NA	NA	−0.01 (−0.19 to 0.17)	.92	−0.03 (−0.10 to 0.03)	.31	−0.01 (−0.19 to 0.16)	.89	−0.03 (−0.10 to 0.04)	.45
**Cardiometabolic risk factors**												
Hypertension												
Past	NA		NA	NA	NA	NA	NA	NA	0.03 (−0.31 to 0.26)	.86	NA	NA
Current	NA		NA	NA	NA	NA	NA	NA	−0.07 (−0.19 to 0.04)	.20	0 (−0.03 to 0.04)	.82
Diabetes												
Past	NA		NA	NA	NA	NA	NA	NA	−0.21 (−0.82 to 0.39)	.49	NA	NA
Current	NA		NA	NA	NA	NA	NA	NA	−0.05 (−0.24 to 0.15)	.63	0.07 (−0.01 to 0.16)	.08

Abbreviations: DSB, Digit Span Backwards; NA, not applicable; PA, Physical Activity.

^a^ Model 1 covariates: No. of missed visits, practice effect (a fixed increment in score from analysis baseline to all following visits), and random effects for intercept and 2 slopes. The maximum No. of women with DSB values in the analysis sample was 1718. Owing to missing values for 1 or more covariates, the sample size for Model 1 was 1640.

^b^ Model 2 covariates: variables in model 1, Study of Women’s Health Across the Nation study site, and each of the demographic characteristics and menopause-associated factors listed in the table. Economic hardship was operationalized as difficulty paying for basic necessities, such as food. The maximum No. of women with DSB values in the analysis sample was 1718. Owing to missing values for 1 or more covariates, the sample size for model 2 was 1582.

^c^ Model 3 covariates: variables in model 2 and cardiometabolic risk factors listed in the table. The maximum No. of women with DSB values in the analysis sample was 1718. Owing to missing values for 1 or more covariates, the sample size for model 3 was 1573.

^d^ The referent woman participated in all Study of Women’s Health Across the Nation cognition visits and had a sport and exercise PA score of 2.77 (scale, 1-5). For models 2 and 3, the referent woman was age 54 years at analysis baseline; was White; had a high school education; took tests in English; reported no financial hardship; did not use hormone therapy; did not report high levels (ie, top quartile) of depressive symptoms, anxiety symptoms, or vasomotor symptoms; and was postmenopausal at analysis baseline. For model 3, the referent woman did not report hypertension or diabetes at any visit.

^e^ For time-varying covariates, past exposure was assessed between Study of Women’s Health Across the Nation cohort baseline and current study analysis baseline (ie, Study of Women’s Health Across the Nation follow-up visit 7). Past exposure was operationalized as the proportion (range, 0 to 1) of visits prior to analysis baseline that the participant reported the exposure. Current values (ie, starting at analysis baseline) of time-varying covariates were modeled to be associated with the outcome of DSB score level at the same visit (ie, contemporaneously), and the value at study baseline was modeled to be associated with the outcome of DSB slope. However, the PA level at each visit was modeled as be associated with the outcome of slope only up to the next visit.

The Supplement provides graphical illustrations of the principal results presented in Tables 2-4: Plots of the model-estimated trajectories are shown for SDMT scores in eFigure 1 in the Supplement, for EBMT-D scores in eFigure 2 in the Supplement, and for DSB scores in eFigure 3 in the Supplement.

Results using total PA level did not differ from those presented for sport or exercise PA levels. We conducted a sensitivity analysis in which we restricted the data to the 4 visits in which both PA and cognition were measured (ie, follow up visits 9, 12, 13, and 15). Sport and exercise PA associations with cognition scores in fully adjusted models remained statistically nonsignificant in these analyses.

## Discussion

This longitudinal cohort study’s aim was to examine whether greater PA during midlife was associated with decreased cognitive aging. We found that cognitive processing speed decreased from the start of the study (when mean age was 51 years) and the rate of decrease accelerated after age 61 years. Verbal episodic memory began decreasing at age 58 years, and working memory began decreasing at age 61 years. In base models, a higher sport and exercise PA level was associated with better concurrent levels of cognitive processing speed and verbal memory and a decreased rate of decline in processing speed and was not associated with the trajectory of working memory. In fully adjusted models, accounting for demographic characteristics, menopause-associated factors, and comorbidities, sport and exercise PA level was not associated with the trajectories of any cognitive test. Neither hypertension nor diabetes were associated with the trajectories of cognitive performance.

Although meta-analyses of more than 2 dozen prospective cohort studies^17,36,37^ found that PA is associated with decreased levels of cognitive decline and dementia, the constraints of the component studies must be acknowledged. In most of these studies, the mean age at which PA was ascertained was 70 years to 80 years, making it plausible that subclinical cognitive decline was associated with an outcome of decreased PA, rather than the converse (ie, reverse causation). Additionally, most cohorts did not use longitudinal analytic methods.^17,36,37^

Ascertaining PA at an earlier life stage may limit concerns about reverse causation, but few studies have done so.^18,34,38,39,40^ In 3 cross-sectional analyses,^38,39,40^ cognitive function was measured once in later life (mean ages ranging from 70-80 years) and its association with self-reported midlife PA was evaluated. In a Swedish twin registry study,^38^ the unadjusted odds of late life dementia were lower in individuals with higher levels of self-reported PA during middle age. Similarly, the adjusted odds of dementia in a Finnish cohort study^40^ were lower among individuals who had previously reported a higher frequency of PA at a mean age of 50 years. In the Study of Osteoporotic Fractures, comorbidity-adjusted results of the Mini-Mental State Exam were 0.1 points to 0.3 points higher and the odds of categorical cognitive impairment were lower among older women who recalled higher levels of PA in their teens, 30s, and 50s.^39^ However, individuals with greater levels of cognitive function may be more likely to exercise; thus, better cognitive function in midlife (not accounted for in these analyses) may explain the findings.^41^ A cross-sectional analysis of repeated waves of data from the Survey of Health, Ageing and Retirement in Europe^34^ found a positive association between self-reported PA level and a concurrently measured composite score on a cognitive test battery. Although this analysis accessed 4 waves of data in a large sample, more than 80% of the analysis sample had only 1 cognition assessment, longitudinal change in cognition was not modeled, and the investigators estimated the association of PA level with current cognition; thus, the results represent repeated cross-sectional associations not longitudinal ones. Our study did not find a longitudinal association between change in PA level and cognitive function over time, a research question which was not addressed by prior cross-sectional studies.

A 2018 longitudinal analysis spanning 28 years from the Whitehall II cohort, which observed participants from midlife to old age, found that PA was not associated with a change in cognitive performance among women or men during midlife.^18^ However, beginning at age 70 years, individuals with higher PA levels had better cognitive trajectories. Furthermore, until 10 years before a dementia diagnosis, the trajectories of PA among individuals who ultimately experienced dementia did not differ significantly from the trajectories of individuals who did not experience dementia. However, starting at 9 years before diagnosis, the PA levels of individuals who eventually experienced dementia were less than those of individuals who did not experience dementia. In aggregate, these findings suggest that reverse causation is of substantive concern when observations begin at older ages. Although our study does not extend into old age, it concurs with the Whitehall II findings of an absence of an association between PA and cognitive trajectories in midlife.

In addition to its PA findings, this study supported prior findings of associations between cognition and sociodemographic characteristics and found new associations in this realm. Concordant with prior work, educational attainment was associated in a graded manner with baseline levels of cognitive performance in all domains but was not associated with the rate of decline in any of these domains.^35,42,43^ Financial hardship (ie, inability to pay for basic necessities) was associated with lower verbal memory levels and greater rates of decline in cognitive processing speed and working memory. Income-associated factors may be associated with cognition by helping to accrue a stronger cognitive reserve, which may mitigate against later declines.^44^ The current study expands this hypothesis, suggesting that financial hardship is associated with lower cognitive reserve (ie, peak function) and more rapid cognitive decline.

Serial assessments of cognitive outcomes and physical activity exposures over a long period are among this study’s principal strengths, permitting use of mixed effects, growth curve modeling. The PA scale inquired about multiple domains, which is important to women’s health because omitting nonsport activities may underestimate women’s PA levels.^45^ Sport and exercise PA level was the primary exposure because it has been used in most cognitive optimization research, but we conducted parallel analyses using total PA level.^4^ Our capacity to limit confounding by menopause-associated symptoms and salient comorbidities is robust. The study sample’s community-based origin and multiethnic/racial composition enhances generalizability. We began observing participants in midlife, filling an information gap in the life course of cognitive aging and minimizing risk of reverse causation.

### Limitations

This cohort study has several limitations, including a small cognitive test battery, a constraint of a multioutcome study. A more difficult or broader test battery might have been more sensitive to decline and its mitigation by PA. For example, frontal brain regions may get preferential benefit from PA; therefore, tests of executive control might have been informative.^46^ While the SDMT is mainly classified as a test of cognitive processing speed and complex attention, it relies on many other domains, including executive function, motor speed, and visuospatial performance, each of which may be associated with PA.^24^ The processing-speed theory of age-associated cognitive decline postulates that because a slower processing speed results in the degradation of multiple cognitive domains, processing speed has a broad influence on cognition.^22^ The annual rates of decline in cognitive performance found in this study were small and may appear inconsequential. However, small longitudinal decline is characteristic of midlife cognitive aging.^47^ We measured PA using the KPAS, which is a self-report measure; it has, however, been validated against objective exercise performance.^31^ Causal inference is constrained in any observational design. However, an RCT of PA beginning in midlife and extending into old age would be impracticable; thus, long-term cohort studies that can undertake longitudinal repeated measure analyses remain an essential component of cognitive preservation research.^18,44^

## Conclusions

This cohort study found that among women at midlife, PA level was not associated with concurrent cognitive scores or a decreased rate of decline in cognitive performance. Most evidence for the hypothesis that PA benefits cognition comes from short-term RCTs or from cohort studies that began at older ages and used cross-sectional analyses. To our knowledge, almost all studies that found a cognitive benefit associated with midlife PA have been cross-sectional. Additional longitudinal cohort analyses that begin in middle age or earlier may help address unanswered questions about whether PA is associated with decreases in longitudinal cognitive decline.

## Notes

Supplement. **eFigure 1.** Model-Predicted Aging Trajectories of Symbol Digit Modalities Test Score **eFigure 2.** Model-Predicted Aging Trajectories of East Boston Memory Test-Delayed Score **eFigure 3.** Model-Predicted Aging Trajectories of Digit Span Backwards Score
