# Supplementary material for: Longitudinal Assessment of Physical Activity and Cognitive Outcomes Among Women at Midlife
Source: JAMA Netw Open. 2021 Mar 31;4(3):e213227. doi: 10.1001/jamanetworkopen.2021.3227 (PMC8013795; doi:10.1001/jamanetworkopen.2021.3227)
Supplement: Supplement. — eFigure 1. Model-Predicted Aging Trajectories of Symbol Digit Modalities Test Score eFigure 2. Model-Predicted Aging Trajectories of East Boston Memory Test-Delayed Score eFigure 3. Model-Predicted Aging Trajectories of Digit Span Backwards Score [file jamanetwopen-e213227-s001.pdf]

Supplemental Online Content

Greendale GA, Han W, Huang MH, et al. Longitudinal assessment of physical activity and cognitive outcomes among women at midlife. *JAMA Netw Open*. 2021;4(3):e213227. doi:10.1001/jamanetworkopen.2021.3227

eFigure 1. Model-Predicted Aging Trajectories of Symbol Digit Modalities Test Score

eFigure 2. Model-Predicted Aging Trajectories of East Boston Memory Test-Delayed Score

eFigure 3. Model-Predicted Aging Trajectories of Digit Span Backwards Score

This supplemental material has been provided by the authors to give readers additional information about their work.

© 2021 Greendale GA et al. *JAMA Network Open.*

eFigure 1. Model-Predicted Aging Trajectories of Symbol Digit Modalities Test Score

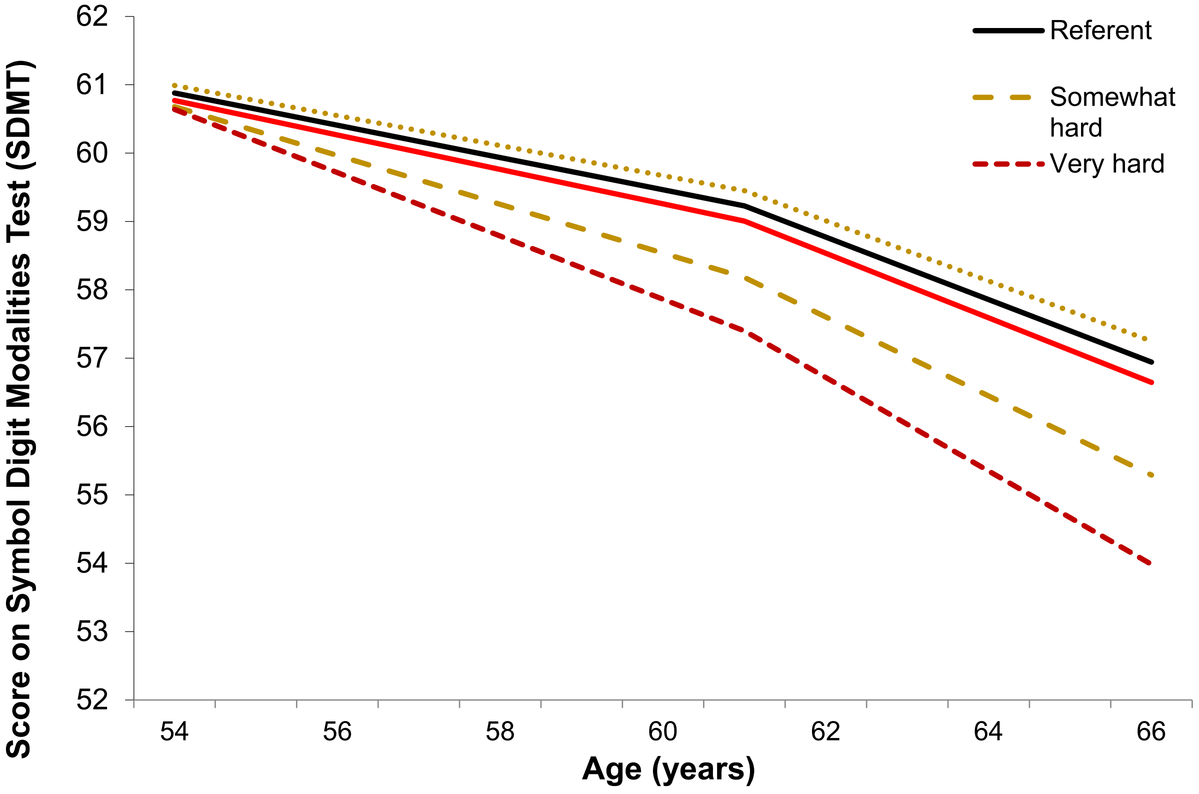

© 2021 Greendale GA et al. *JAMA Network Open.*

Legend eFigure 1

Model-predicted trajectories of Symbol Digit Modalities Test scores (range 0-110) in the following women. The referent woman, who participated in all SWAN cognition visits, had sport/exercise physical activity score of 2.77 [1 to 5 scale], aged 54 years at analysis baseline, of White race/ethnicity, had high school education, took tests in English, reported no financial hardship, did not use hormone therapy, did not report high levels [top quartiles] of depressive symptoms, anxiety symptoms, and vasomotor symptoms, was postmenopausal at analysis baseline, and did not report hypertension or diabetes at any visit. Women who differed from the referent woman only in that they were more or less physically active, defined as scoring higher or lower by 1 point on the sport/exercise scale. Women who differed from the referent woman only with respect to financial hardship, reporting that it was somewhat hard to pay for basics or very hard to pay for basics.

© 2021 Greendale GA et al. *JAMA Network Open.*

eFigure 2. Model-Predicted Aging Trajectories of East Boston Memory Test-Delayed Score

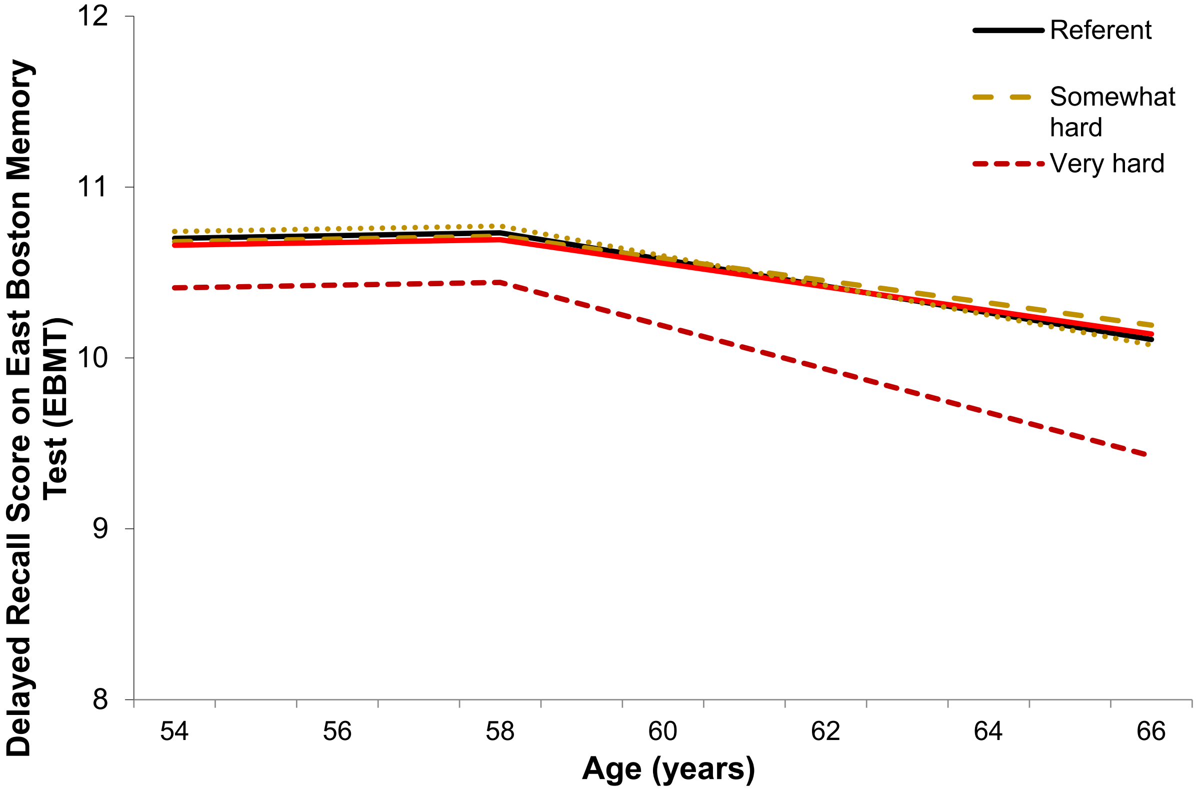

© 2021 Greendale GA et al. *JAMA Network Open.*

Legend eFigure 2

Model-predicted trajectories of East Boston Memory Test-delayed recall (EBMT-delayed) scores (range 0-12) in the following women. The referent woman, who participated in all SWAN cognition visits, had sport/exercise physical activity score of 2.77 [1 to 5 scale], aged 54 years at analysis baseline, of White race/ethnicity, had high school education, took tests in English, reported no financial hardship, did not use hormone therapy, did not report high levels [top quartiles] of depressive symptoms, anxiety symptoms, and vasomotor symptoms, was postmenopausal at analysis baseline, and did not report hypertension or diabetes at any visit. Women who differed from the referent woman only in that they were more or less physically active, defined as scoring higher or lower by 1 point on the sport/exercise scale. Women who differed from the referent women only with respect to financial hardship, reporting that it was somewhat hard to pay for basics or very hard to pay for basics.

© 2021 Greendale GA et al. *JAMA Network Open.*

eFigure 3. Model-Predicted Aging Trajectories of Digit Span Backwards Score

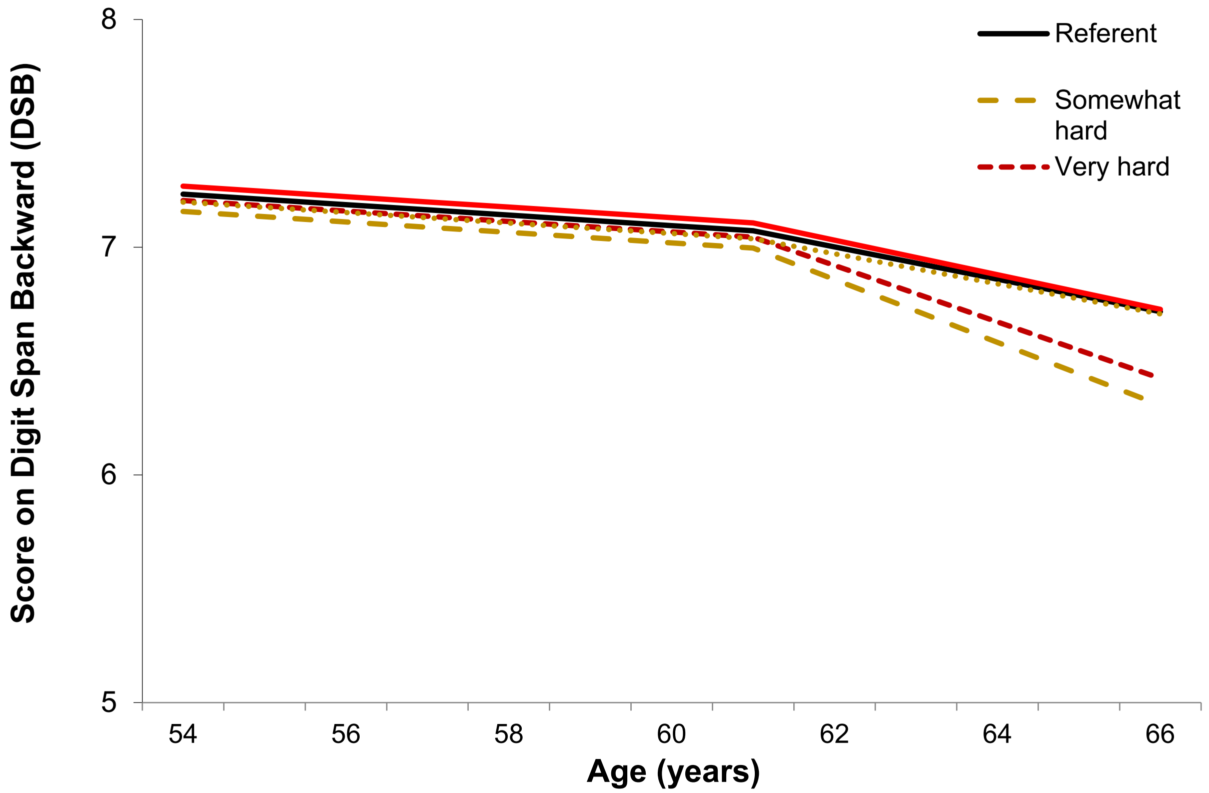

© 2021 Greendale GA et al. *JAMA Network Open.*

Legend eFigure 3

Model-predicted trajectories of Digit Span Backwards (DSB) scores (range 0-12) in the following women. The referent woman, who participated in all SWAN cognition visits, had sport/exercise physical activity score of 2.77 [1 to 5 scale], aged 54 years at analysis baseline, of White race/ethnicity, had high school education, took tests in English, reported no financial hardship, did not use hormone therapy, did not report high levels [top quartiles] of depressive symptoms, anxiety symptoms, and vasomotor symptoms, was postmenopausal at analysis baseline, and did not report hypertension or diabetes at any visit. Women who differed from the referent woman only in that they were more or less physically active, defined as scoring higher or lower by 1 point on the sport/exercise scale. Women who differed from the referent woman only with respect to financial hardship, reporting that it was somewhat hard to pay for basics or very hard to pay for basics.

© 2021 Greendale GA et al. *JAMA Network Open.*
